# Adolescents With Chronic Medical Conditions and High School Completion: The Importance of Perceived School Belonging

**DOI:** 10.5334/cie.5

**Published:** 2020-04-27

**Authors:** Kathryn M. Kirkpatrick

**Affiliations:** 1 Nationwide Children’s Hospital, US

**Keywords:** adolescents, school belonging, chronic medical conditions, high school completion

## Abstract

Students with chronic medical conditions often experience barriers to academic progress, including impact of disease and treatment, increased school absence, and altered expectations of teachers and parents. School belonging is an important element of academic success and can be promoted by positive relationships, structure, and support in the school environment. One aim of this study was to explore group differences in perceived school belonging and rate of on-time high school completion for students with chronic medical conditions as compared to their healthy peers. The second goal was to analyze relations between belonging, health status, and on-time completion of high school. Restricted data from Add Health was used to answer the study questions.

Results showed that students with chronic medical conditions reported lower levels of perceived school belonging than their healthy peers (*t*_(1056)_ = 3.69, *p* < .001, *d* = 0.23). Students with chronic medical conditions also attained lower levels of on-time high school graduation than their healthy peers (*t*_(1056)_ = 2.60, *p* = .005, *d* = 0.16). Perceived school belonging had a different impact for students with chronic medical conditions than for those who had no health concerns. Each unit increase in belonging for students with health impairment was related to a 63% increased likelihood of on-time high school graduation (OR = 1.629, *p* = .003). School belonging is especially important for students living with chronic medical conditions. Finding ways to facilitate a stronger sense of school belonging may be a way to support desired academic outcomes.

As many as 20% of the school-age youth in the United States live with a chronic medical condition and about one third of those students experience sequelae severe enough to interfere with school participation on a regular basis (Kaffenberger, 2006; Shaw, Glaser, Stern, Sferdenschi, & McCabe, 2010; Thies, 1999). These numbers mean that, across their careers, any classroom teacher or school administrator is likely to encounter multiple children managing a chronic medical condition, generally defined as a “medical condition, lasting for more than 3 months, which requires medical attention and interferes with a person’s daily living” (Barraclough & Machek, 2010, p. 132). Chronic medical conditions impact both physical and psychological functioning for a child in domains that include family, school, and peer relationships (Brown & Anderson, 1999; Power, 2006; Shaw et al., 2010).

Some researchers have suggested the use of a non-categorical approach when studying the social or educational impact of illness for young people (Perrin et al., 1993; Stein, Bauman, Westbrook, Coupey, & Ireys, 1993), meaning that all students have many common life experiences, despite the actual diagnosis involved (Davis & Brosco, 2007; Perrin et al., 1993; Stein et al., 1993; Stein & Jessop, 1989). Thus, different disease processes may have different mechanisms of impact, but the resulting challenges in a school setting look similar across populations. For example, educators can accommodate slowed cognitive processing, fatigue, pain, or inattention with appropriate intervention strategies regardless of the origin of the difficulty.

Evidence suggests that children and adolescents who experience neurological or cognitive impairment as a part of their condition have more difficulty than those who do not have such sequelae (Howe, Feinstein, Reiss, Molock, & Berger, 1993; Northam, 1997). However, Howe and colleagues (1993) concluded that though children with brain-based limits have different risks and needs than children with other consequences of their medical condition, even children with those more general impacts fare more poorly overall than their healthy peers.

## Impact of Chronic Medical Conditions at School

When young people must balance the demands of managing a chronic medical condition along with the school demands all adolescents experience, a new dimension is added to the quest for academic and social success. There is no reason to believe that adolescents with chronic medical conditions would have any less desire than their healthy peers to meet their basic psychological and developmental needs (Schwartz & Drotar, 2009; Shaw & McCabe, 2008; Svavarsdottir, 2008). However, depending on the level of impact a given condition has on the physical, emotional, or cognitive aspects of the student’s life, he or she may experience interruptions to typical developmental and academic progress (Fowler, Johnson, & Atkinson, 1985; Northam, 1997; Schwartz & Drotar, 2009; Shaw et al., 2010).

Effects of health on academic performance may be directly related to the cognitive impact of disease and treatment or a result of indirect factors (Haas, 2006; Lum et al., 2017; Madan-Swain, Fredrick, & Wallander, 1999). Factors that can directly affect academic outcomes include fatigue, pain, and cognitive changes resulting from the disease and treatment. Indirect effects arise via school attendance patterns and related missed instructional time, changes in teacher and parental expectations for academic achievement, or other psychosocial adjustment issues such as the development of school phobia or separation anxiety (Clay, Cortina, Harper, Cocco, & Drotar, 2004; Haas, 2006; Jackson, 2009; Maslow, Haydon, Ford, & Halpern, 2011; Sexson & Madan-Swain, 1995).

Academic struggles may occur acutely as a result of disease symptoms and treatment side effects or become evident later, when the child reaches an age at which the affected skills would be expected to emerge developmentally (Armstrong, 2006; Armstrong & Briery, 2004). Acute interference with academic progress is often a result of pain, fatigue, lethargy, general malaise, or medication side effects (Madan-Swain et al., 1999; Meijer, Sinnema, Bijstra, Mellenbergh, & Wolters, 2000; Thies, 1999). When cognitive effects appear over time, they do not represent deterioration of previously accomplished milestones, but are the result of impact on the rate of brain growth and development of complex structures within the brain (Armstrong & Briery, 2004; Daly, Kral, & Brown, 2008; Moore, 2005, Oeffinger, Nathan, & Dremer, 2008). Neurocognitive sequelae can include impact on cognitive ability, attention, processing speed, memory, visual-motor integration, school performance, social interactions, and adaptive behaviors (Armstrong & Briery, 2004; Brown & Anderson, 1999; Burke & Elliott, 1999; Martinez & Ercikan, 2008; Meijer et al., 2000; Power, 2006). These neurocognitive deficits, in turn, may interfere not only with academic progress but also with the development of competence and social skills (Power, 2006). They may be observed by parents and by teachers in the classroom as failure to complete work, slowness with approaching and accomplishing work, periods of inattention, fine-motor deficits, or difficulty with developing and maintaining peer relationships (Armstrong & Briery, 2004; Madan-Swain, Katz, & LaGory, 2004).

## Academic Attainment

Children with a variety of chronic medical conditions have demonstrated lower levels of academic attainment, even when they appear to have typical cognitive functioning or global IQ scores similar to those of healthy peers (Berg & Linton, 2009; Case, Fertig, & Paxson, 2005; Fowler et al., 1985; Haas & Fosse, 2008; Sexson & Madan-Swain, 1993, 1995). Evidence suggests that children and adolescents with chronic medical conditions reach developmental milestones later than their healthy peers (Barraclough & Machek, 2010; Maslow et al., 2011; Suris, Michaud, & Viner, 2004) and are less likely to complete high school on time (Champaloux & Young, 2015; Haas & Fosse, 2008; Maslow, Haydon, McRee, & Halpern, 2012). These lower levels of academic attainment have consequences for vocational achievement and psychosocial outcomes in adulthood (Case et al., 2005; Joe, Joe, & Rowley, 2009; Maslow et al., 2012; Needham, Crosnoe, & Muller, 2004).

## School Belonging and Connectedness

Belonging has been defined as a basic human need, important for optimal functioning (Baumeister & Leary, 1995; Goodenow, 1993; Libbey, 2004). Humans need an integrated and reciprocal relationship with others in the community. For children and adolescents, school is the primary community outside the family. School environments with characteristics such as high academic standards, high levels of teacher support, a community where relationships between students and adults are caring and respectful, and safety have been identified as promoting school connectedness for students (Blum, 2005; Libbey, 2004; Wingspread Conference, 2004) and as being important for students of all ages (Anderman, 2003; Anderman & Freeman, 2004; Birch & Ladd, 1997). Many benefits of school belonging for student success have been noted, including social, behavioral, and academic (Anderman & Anderman, 1999; Blum, 2005; Goodenow, 1993; Libbey, 2004; Wingspread Conference, 2004).

Not surprisingly, school connectedness has been identified as a protective factor for adolescents (McNeely, Nonnemaker, & Blum, 2002; Resnick et al., 1997), and it may be especially related to academic attainment for students with chronic medical conditions (Lum et al., 2017; Maslow et al., 2011). Social isolation can be a greater concern for students with medical conditions, and it may be that lack of connectedness is related to lost opportunities for development of social relationships. Peer interaction and teacher-student relationships may be especially important for students with chronic medical conditions, as those relationships can foster a sense of school belonging just as they do for healthy, typical students (Capurso & Dennis, 2017; LaGreca, Bearman, & Moore, 2004; McMahon, Parnes, Keys, & Viola, 2008; Shiu, 2001). When youngsters feel they belong, they may be able to pull from a stronger set of inner resources. Conversely, they may suffer when they anticipate isolation from experiencing decrements in reasoning and thought processing (Baumeister, Twenge, & Nuss, 2002).

A sense of school belonging seems to have long-term implications for adolescents (Eccles et al., 1993; Finn, 1989; Patrick, Anderman, & Ryan, 2002). That is, when students experience a sense of belonging or connectedness to their school and its values, they are likely to have positive school functioning and are less likely to leave school before graduation (Finn, 1989; Murdock, 1999). Young adolescents need an opportunity to interact with peers, teachers, and a mastery-oriented environment so they can become invested and motivated for academic tasks (Anderman & Freeman, 2004).

Students with chronic health conditions have consistently described lower levels of motivation, engagement, school satisfaction, feelings of safety, and academic achievement along with higher levels of loneliness and isolation compared to healthy peers (Forrest Bevans, Riley, Crespo, & Louis, 2011; Hogan, McLellan, & Bauman, 2000; McDougall, DeWit, King, Miller, & Killup, 2004; Svavarsdottir, 2008). When adolescents with chronic medical conditions are absent from school frequently or have low levels of energy, they are likely to have decreased levels of both instructional and social participation in the community and can develop a sense of isolation or experience academic struggle (Needham et al., 2004; Phelps, 2006; Thies, 1999). High-risk students may need to be offered specific opportunities for positive teacher and peer relationships in an effort to encourage a sense of school belonging.

## Present Study

The primary aim of the current study was to examine associations between an adolescent’s health status, perceptions of school belonging, and the likelihood of high school completion by 19 years of age. The following four questions guided the study:

1. *Does health status predict level of perceived school belonging in adolescents?*
  It was hypothesized that poorer health status (as measured by health reports and a symptoms score) would be associated with lower student perceptions of school belonging.
2. *Are there mean differences in the levels of perceived school belonging between adolescents who have had a chronic medical condition since childhood and healthy peers?*
  It was hypothesized that students with chronic medical conditions would report lower levels of perceived belonging.
3. *Does perceived school belonging predict the odds of high school completion by the age of 19 years?*
  It was hypothesized that higher levels of perceived school belonging would be related to higher high school completion rates.
4. *Is the relation between perceived school belonging and high school completion the same for adolescents with chronic medical conditions since childhood and their peers who report no health concerns?*
  It was hypothesized that health status would moderate the association between perceived belonging and high school completion.

## Methods

The National Longitudinal Study of Adolescent to Adult Health (Add Health; Harris et al., 2009) is a long-term study using a nationally representative sample of U.S. adolescents who were in grades 7–12 during the 1994–95 school year. A stratified, complex sample was used, and five waves of data have been collected to date. The purpose of the Add Health project has been to explore the social, economic, psychological, and physical outcomes for the participants in a variety of contexts as they moved from adolescence into adulthood (Carolina Population Center, n.d.).

Wave I data were collected via in-home interviews of both parents and students in the 1994–95 school year. Wave III data were collected in 2001–02, when the participants were 18 to 26 years old. Wave IV data were collected in 2007–08 when participants were 24 to 32 years old Waves III and IV included in-home interviews of all participants from Wave I who could be located.

Wave II of Add Health used a different set of participants, and is not included in this study. Wave V data have not yet been released. Waves I, III, and IV were each used to gather particular pieces of data for the current study. Table 1 provides a description of the Add Health information used in this study. Waves III and IV were used when the variable of interest was not available in an earlier wave of data.

**Table 1:** Participants and Information Included in Add Health Waves I, III, and IV.

	Students		Parents	Information Used in Current Study

Wave	Core Sample	Oversamples		

**Wave I** In-Home Interview 1994–95	Random selection from stratum of US students in grades 7–12 (*N* = 12,105)	Purposeful selection for oversamples plus saturation samples of 16 schools (*N* = 8,640)	Parents of participating students (*N* = 17,670)	* Demographic variables * Health report * Items for measurement of perceived belonging * Grades * Report of symptoms
	Total in-home interview sample of grade 7–12 students at Wave I (*N* = 20,745)			
**Wave III** In-Home Interview 2001–02	All Wave I respondents (now aged 18–26 years) who could be located and re-interviewed (*N* = 15,197)			* Information about on-time high school completion
**Wave IV** In-Home Interview 2007–08	All Wave I respondents (now aged 24–32 years) who could be located and re-interviewed (*N* = 15,701)			* Information related to diagnosis of chronic medical conditions in childhood * Student level weights

SPSS 20 (IBM Corp, 2011) software was used for initial data cleaning and recoding. Stata 12 (StataCorp, 2011a) software was used to compute the results of all further analyses. The Stata 12 survey set commands address the stratified, clustered and weighted nature of the data (StataCorp, 2011b) and allow for accurate parameter and variance estimates. Design-based features, including stratum, cluster, and weights, were included in analyses, as appropriate (Chantala, 2006).

### Participants

Participants from Wave I of the Add Health study who had complete data for the items of interest (*N* = 20,180) were included for variable development. Wave III data were used to determine which participants from Wave I had completed high school by the age of 19. Wave IV data were used to determine which Wave I participants identified the diagnosis of a chronic medical condition prior to the age of 12 years. Wave IV data were also used for a logistic regression model to assess the likelihood of on-time high school completion based on health status and levels of perceived school belonging.

Subsamples were identified from the larger Add Health sample for the purpose of assessing mean levels of perceived school belonging and rate of high school completion by age 19 based on health history. Two groups were chosen based on self-reported information about diagnosis of a chronic medical condition collected at the Wave IV data time point.

The first group included participants who reported diagnosis of a chronic medical condition at the age of 12 years or younger (*N* = 629). Diagnoses in the chronic medical condition group included cancer (*n* = 27), diabetes (*n* = 40), heart disease (*n* = 44), epilepsy (*n* = 107), and migraine (*n* = 436). These conditions have been noted to have similar impact on school (Barrett & Sachs, 2006; Berg & Linton, 2009; Brown, 2004; Clay, 2004; Delamater, Brady, & Blumberg, 2004; Phelps, 2006; Powers, Patton, Hommel, & Hershey, 2003; Robinson et al., 2010; Rovet & Fernandes, 2004; Taras & Potts-Datema, 2005) and multiple previous studies have used a similar list of diagnoses with the non-categorical approach for research (Cadman, Boyle, Szaltmari, & Offord, 1987; Fowler et al., 1985; Maslow et al., 2011; Meijer et al., 2000; Thies, 1999). The healthy group (*N* = 897) was randomly chosen from all who identified as having no history of health concerns at the Wave IV data collection point. Table 2 provides information about the characteristics of each of the groups in the study.

**Table 2:** Characteristics of Participants in the Study.

Variable	Range	WAVE I Full Data Set (*N* = 20,180)		Subsamples		Wave IV Full Data Set (*N* = 10,813)

		Design-Based *M* (se)	Unweighted *M (SD)*	Healthy (*N* = 897) *M (SD)*	Chronic Ill (*N* = 629) *M (SD)*	Design-Based *M* (se)

Age	12–19	15.85 (.12)	16.11 (1.70)	16.07 (1.73)	16.0 (1.69)	15.82 (.12)
Gender	%female	50.5%	50.5%	47%	63%	52%
Race	%minority	27.9%	42.3%	42%	32%	27%
Parent Educ	1–5	2.67 (.05)	2.68 (1.14)	2.71 (1.14)	2.59 (1.07)	2.76 (.05)
Grade Avg	1–4	2.85 (.02)	2.75 (.77)	2.79 (.77)	2.72 (.76)	2.90 (.02)
Belong Scale	1–5	3.71(.02)	3.68 (.75)	3.74 (.71)	3.59 (.82)	3.74 (.02)
Health Report	1–5	4.01 (.01)	3.97 (.76)	4.06 (.73)	3.74 (.82)	4.03 (.02)
Symptoms Score	0–31	0.78 (.01)	0.76 (.45)	7.70 (4.50)	10.60 (5.78)	0.77 (.01)
Complete HS	%Yes	92.7%	92.3%	94.4%	90.1%	93.7%

*Note*: Gender: 0 = male, 1 = female; race: 0 = white, 1 = minority; HS completion: 0 = No, 1 = Yes.

### Measurement

Control variables were developed using the Wave I Add Health data. Age, gender, and race were all available in the Wave I data. Grade average was computed by using the mean of the most recent grades for English, math, science, and history reported by the participant. The parent education variable was created using the mean of mother and father education level when both were reported. The completion level of one parent was used when only one was identified.

#### Health report at Wave 1

Students and their parents were each asked to assess the student’s general health at Wave 1 (“*In general, how is your/student’s health?*”; scale of 1 = poor to 5 = excellent). The health report value was created using the mean of the two scores reporting health status. Many chronic medical conditions are not identified in the Add Health data, so the health report at Wave 1 was added as a more general health variable for the assessment of outcomes.

#### Symptom score

Students reported the frequency of a variety of physical symptoms for the previous 12 months. Responses ranged from 0 (never) to 4 (almost every day) for each symptom. A mean score indicating the self-reported frequency of 11 symptoms was developed from Wave1 responses. The items describe a variety of student complaints, some that would be considered more physical (i.e., headaches, feeling weak) and others that might be categorized as more emotional (i.e., moodiness, unable to relax). Physical and emotional symptoms were both included as a way of addressing the fuzziness that exists between the two, and because the symptoms, per se, were not the focus of this study.

Principal-components analysis with an oblique rotation was used to assess the items. The symptoms loaded on one factor with an eigenvalue of 3.59. The 11 items had an alpha coefficient of 0.79. The variable was created by using a composite symptoms mean score (range 0–31; *M* = 0.78, linearized se = .01). This variable was also used as a general measure of student health for the initial analyses.

#### Perceived school belonging

A scaled score was created using five items from the Wave I student survey: *You feel close to people at your school; You feel like you are part of your school; You are happy to be at your school; The teachers at your school treat students fairly*; and *You feel safe in your school*. The items were measured using a 5-point Likert scale, ranging from 1 = strongly disagree to 5 = strongly agree, and the scale was created using the average of responses for each of the items (*M* = 3.71, linearized se = .02). Previous researchers have used the same items to assess the construct of school belonging (Anderman, 2002; Maslow et al., 2012; McNeely et al., 2002; Resnick et al., 1997). The 5-item scale had an acceptable level of reliability for the current sample (α = .76).

#### High school completion

Age of completion was calculated using participant date of birth and Wave III responses about high school graduation or GED completion dates. This outcome variable was a binary measure (yes or no). Completion by the age of 19 years was considered “on-time” completion for the purposes of this study.

## Results

### Prediction of Perceived Belonging

Linear regression modeling with Wave I data was used to explore the relation between levels of perceived belonging and health variables. Gender, race, and parent education were not significant predictors of belonging in the final regression model. Age was inversely related and grade average was positively related, and both were significant predictors of perceived belonging. Report of health status had a positive relationship with belonging, while symptom scores had an inverse relation with belonging. Health report and symptoms score both provided significant predictive utility for perceived belonging. With demographic variables controlled, the two health variables together explained an additional 7.2% variance for perceived belonging. Final regression model values are found in Table 3.

**Table 3:** Final Regression Model for Perceived School Belonging Using Design-Based Wave I Data.

	Stand. Coefficient	Linearized se	*p*

Age	–.029	.008	<.001
Gender	.001	.020	.947
Race	–.026	.025	.307
Parent Education	–.009	.009	.344
Grade Average	.197	.014	<.001
**Initial Model R^2^**	**.067**		
Wave I Health Report	.120	.015	<.001
Symptoms Mean Score	–.377	.027	<.001
**Model R^2^△**	**.072**		
**Total Model R^2^**	**.139**		

*Note*: Gender coded male = 0, female = 1; race coded white = 0, minority =1; bold = variance explained by control variables, additional variance explained by health variables and total variance explained.

### Group Differences

T-tests were used to examine mean differences for the demographic variables between the group of students with chronic medical conditions and the group of healthy peers (total *N* = 1,526). Hedges *g* was used for effect size estimation (Fritz, Morris, & Richler, 2012).

Based on diagnostic status (yes vs. no), no differences were found for age or prior grades. The reported parent level of education was higher for the healthy group of participants (*t*_(1464)_ = 2.10, *p* = .014, *g* = 0.11). The chronic medical condition group included more females (*t*_(1552)_ = –6.32, *p* < .001, *g* = 0.33). The healthy group included more students of minority status (*t*_(1549)_ = 4.17, *p* < .001, *g* = 0.22).

Results of previous research have indicated that gender classification may lead to differing levels of chronic medical condition incidence and consequences of the disease and treatment (Berg & Linton, 2009; Case et al., 2005; Madan-Swain et al., 2004). The differences in minority membership may be related to the oversampling for the Wave I data collection. All of the differences had a small effect size.

A structured mean differences model was developed to assess participants’ understanding of the belonging construct across the two groups. Structured mean differences models are used to determine whether the latent (unobservable) construct is perceived similarly by individuals in both groups. The latent construct (belonging) is measured using observable variables (items from survey). Model fit is the match between the covariance matrix of the groups and the estimated covariance matrix of the population. In a good fit, the two groups would both match the population matrix as closely as possible (Acock, 2013; Schumacker & Lomax, 2010).

The groups were assessed for invariance, and the final model indicated that the two groups perceived the concept of belonging in a similar fashion. Model fit statistics are presented in Table 4. Goodness-of-fit parameters may be found in Table 5.

**Table 4:** Model Comparisons for Healthy and Chronically Ill Student Groups.

Model	*X* ^2^	*df*	*p*	*X*^2^ difference**	*df* difference	Significance (difference)	RMSEA	CFI	SRMR

1. Same Form	301.71	98	<.001	–	–	–	.052	.974	.028
2. Same Loadings	302.07	106	<.001	.36_(1v2)_	8	ns	.049	.975	.032
3. Same Loadings and Err Variances	407.17	133	<.001	105.10_(2v3)_	27	Sig.	.052	.965	.051
4. Same Loadings and Intercepts	341.29	119	<.001	39.22_(2v4)_	13	Sig.	.049	.972	.039

*Note*: Target parameters for good model fit are RMSEA < .05, CFI > .95, and SRMR < .05; RMSEA = root mean square error of approximation, CFI = comparative fit index, SRMR = standardized root mean-squared residual. The goal for *X*^2^ is non-significance; however, large sample size may lead to a significant *X*^2^ result. ** Model elements are constrained to compare between the groups, 1 v. 2 = comparison between fully unconstrained model and model with constrained factor loadings, 2 v. 3 = comparison between constrained loadings only and constrained loadings plus error variances, 2. v. 4 = comparison between constrained loadings only and constrained loadings and intercepts.

**Table 5:** Group Goodness-of-Fit Parameters (SRMR) for Comparison Models.

	Observations	1. Same Form	2. Same Loadings	3. Same Loadings and Error Variances	4. Same Loadings and Intercepts

Healthy	924	.024	.027	.046	.034
Chronically Ill	627	.032	.037	.055	.044

*Note*: SRMR = standardized root mean-squared residual, target < .05.

#### Perceived belonging

Group mean levels of perceived school belonging were compared using a *t*-test (healthy vs. chronically ill). The healthy group had a mean of 3.78 (*SD* = 0.68), whereas the group with chronic health conditions had a mean of 3.61 (*SD* = 0.80). The mean difference was significantly different than zero (*t*_(1056)_ = 3.687, *p* < .001), with an effect size in the small range (*d* = 0.23; Cohen, 1988). As a result, the hypothesis stating that healthy students would report higher levels of belonging than those with chronic medical conditions was supported.

#### High school completion

Group differences in the rate of high school graduation by age 19 were significantly different than zero (*t*_(1056)_ = 2.60*, p* = .005*)* with a small effect size (*d* = 0.16; Cohen, 1988). Healthy students had an on-time high school completion rate of 94.4% (*M* = 0.9439, *SD* = 0.230), whereas students with chronic medical conditions had on-time high completion rates of 90.1% (*M* = 0.9014, *SD* = 0.298). This finding supports the hypothesis that healthy students would have higher high school completion rates than their chronically ill peers.

### Belonging and High School Completion

After the differences in mean levels of perceived belonging and high school completion were established, logistic regression was used to determine the relation of belonging and diagnostic status to the likelihood of on-time high school completion. For the logistic regression model, the full Add Health data set was used with Wave IV weights applied (Chantala, 2006). Age, gender, race, parent education, and prior grades were all significant predictors of high school completion by the age of 19. Level of perceived belonging was a significant predictor of on-time high school completion as well. Diagnostic status did not have a significant main effect on the outcome, but it did serve as a significant moderator for belonging. Holding all demographic variables constant, perceived belonging had a different relation with likelihood of on-time high school completion for a student with a chronic health condition than for a peer without an identified health condition.

While belonging is important for all students, those with identified medical conditions experienced more benefits from increased levels of perceived belonging than their healthy peers. The odds ratios for each of the variables are included in Table 6. As illustrated, the odds ratio for the moderation of diagnosis status on belonging was 1.629 (*p =* .003), indicating that an increase of one unit in perceived belonging led to a 63% increase in the odds of completing high school on time for a student with a chronic medical condition. An odds ratio of this magnitude is considered to have a small effect size (Sullivan & Feinn, 2012).

**Table 6:** Design-Based Coefficients and Odds Ratios for Final Logistic Regression Model to Predict High School Completion by the Age of 19 Years (N = 10,253).

Variable	*b* (log odds) (Linearized se)	*p*	OR (Linearized se)	95% CI for OR

Age	–.3252 (.034)	<.001	.7223 (.024)	.6759, .7719
Gender	.3185 (.123)	.011	1.3751 (.169)	1.0786, 1.7530
Race	–.4326 (.146)	.004	.6488 (.095)	.4861, .8659
Parent Education	.5008 (.071)	<.001	1.6500 (.117)	1.4342, 1.8983
Grade Average	.9250 (.076)	<.001	2.5219 (.192)	2.1697, 2.9313
W1 Health Report	.1703 (.078)	.031	1.1857 (.093)	1.0158, 1.3840
Diagnosis Status	–.5055 (.751)	.502	.6032 (.453)	1.1365, 2.6651
School Belonging	–.3373 (.130)	.010	.7137 (.092)	.5523, .9222
Diag X Belonging	.4882 (.158)	.003	1.6293 (.258)	1.1911, 2.2287

*Note*: High school completion coded no = 0, yes = 1; Gender coded 0 = male, 1 = female; Race coded 0 = white, 1 = minority; diagnosis coded 0 = no, 1 = yes

## Discussion

The findings of this study contribute to the fields of education and psychology in several ways. Specifically, helping school staff (teachers, administrators, coaches, etc.) understand the importance of perceived belonging for all students under their charge may be a way to promote academic attainment. Students need to reach the first level of academic attainment – high school completion – before they can move on to higher levels of attainment.

It is critical for school personnel to understand that belonging is important for all students, but especially for students with chronic medical conditions or other or identified health concerns. If health status and belonging interact to provide some explanation of variance for academic attainment, it seems prudent for academic leaders to find ways to promote perceived belonging for students with chronic medical conditions/health concerns and to create a supportive environment for high school completion.

There are several ways the findings reported here can be addressed in the school setting. For example, acknowledging the extra struggle that a student with a chronic medical condition may experience and communicating empathy can promote perceived belonging. Further, school personnel can strive to create a safe environment for all students, particularly those who sometimes feel different because of frequent absence or visible signs of a medical condition. In addition, expressing awareness of the issues experienced by students with chronic medical conditions that might not be obvious (e.g., fatigue, processing speed delays) could support a positive relationship with the students.

### Relationships

Teacher-student relationships have been shown to have a clear positive impact on students’ sense of school belonging (Goodenow, 1993; Klem & Connell, 2004; Libbey, 2004; McNeely & Falci, 2004; Murray & Greenberg, 2000; Patrick et al., 2002; Rosenfeld, Richman, & Bowen, 2000; Wentzel, 1998). Teachers can impact their students through caring, treating the them fairly, and actively engaging them in learning. For example, Libbey (2004) noted that “student relationships with their school often were operationalized as their relationship with their teachers” (p. 281). Similarly, Goodenow (1993) found that teacher support explained more than one third of students’ assessment of value and interest related to their academic work.

Pedagogical caring (Klem & Connell, 2004; Noddings, 1992; Wentzel, 1997) is a concept that involves the ways that students perceive care from their teachers. Teacher behaviors that suggest pedagogical caring include modeling caring behaviors, democratic communication style, treating students as individuals, structure and expectations, and a nurturing manner. When students feel cared for by their teachers, they are more likely to be academically motivated and pursue prosocial goals (Klem & Connell, 2004; Wentzel, 1997). Capurso and Dennis (2017) identified relationships (including authentic social interactions with peers and teachers) as one of six Key Educational Factors (KEF) necessary for students with chronic medical conditions to have a successful school experience.

There is research available to provide guidance to school administrators and teachers about how to provide belonging support for students. The Wingspread Declaration (Wingspread, 2004) describes school connection as “the belief by students that adults in the school care about their learning as well as about them as individuals” (p. 233). Three critical school-level components of belonging are identified as predictors of positive academic outcomes for students: (a) high academic expectations coupled with support for learning, (b) positive and respectful student-adult relationships, and (c) physical and emotional safety of the school environment (Blum, 2005; Klem & Connell, 2004; Wingspread, 2004).

## Conclusion

The findings of this study support prior reports that students with chronic medical conditions experience more difficulty in the school environment than their healthy peers. The hypothesis that students with chronic medical conditions would be less likely to complete high school on time was supported. The hypothesis that students with chronic medical conditions would experience lower levels of perceived school belonging was also supported. The important finding that perceived school belonging is necessary for students, especially those with chronic medical needs, should be kept in mind as school administrators and teachers look for ways to promote high rates of high school completion for all students.

### Limits of the Study

Limitations come with any study using secondary data. Some of the concerns related to this study include the abbreviated list of diagnoses identified in the Add Health data set, small numbers for any one specific disease group, and the lack of information about the intensity of disease for the participants. It is also important to note that the diagnosis of a chronic medical condition before the age of 12 was self-reported by the participants when they were between 24 and 32 years old. Many of the more common pediatric chronic conditions were excluded from the list, and those exclusions may have made the division between healthy and chronically ill students blurry and difficult to account. For example, students with sickle cell disease or inflammatory bowel disease are not identified in the Add Health sample but are among the students with significant care needs that can interfere with school (Brown, 2004; Clay, 2004; Daly et al., 2008; Phelps, 2006). Large data sets in the United States are typically focused on health or education factors, and both domains are rarely included in the same study. Therefore, some of the detail of either domain may be lost when the focus is on the other.

Further, there are pros and cons of using a non-categorical approach to the study of students with chronic medical conditions. Hundreds of pediatric diseases could be considered, so the purpose of the study is important to keep in mind. While a non-categorical approach does not allow for fine-grained detail related to any one specific diagnosis, it can move the understanding of a students with a broader range of diagnoses forward and allow for more general guidance around school policies and practices.

### Implications for Further Research

Further study of a larger and more heterogeneous group of students with chronic medical conditions will be useful in understanding ways to address student need for belonging. Any study that looks at the impact of chronic medical conditions at school should include not only identification of diagnosis but also an indicator of disease or symptom severity. Perhaps severity of impact (pain, fatigue, school absence, social isolation) is more important for school personnel to understand than a focus on specific diagnosis.
